# Food intake attenuates the drug interaction between new quinolones and aluminum

**DOI:** 10.1186/s40780-018-0107-1

**Published:** 2018-05-14

**Authors:** Ayuko Imaoka, Kosuke Abiru, Takeshi Akiyoshi, Hisakazu Ohtani

**Affiliations:** Division of Clinical Pharmacokinetics, Keio University Faculty of Pharmacy, 1-5-30, Shibakoen Minato-ku, Tokyo, 105-8512 Japan

**Keywords:** Drug - drug interaction, Absorption, Food, New quinolones, Ofloxacin, Ciprofloxacin, Aluminum, Chelation

## Abstract

**Background:**

Intestinal absorption of new quinolones is decreased by oral administration of polyvalent metal cations. Some clinical studies have demonstrated this drug - drug interaction is more prominent under fasted condition. However, the effect of food intake on the extent of drug - drug interaction between new quinolones and metal cations remains to be investigated quantitatively and systematically. The aim of this study was to develop an animal model that enables to evaluate the effect of food intake on the extent of drug - drug interaction in the gastrointestinal tract by chelation and to apply the model to evaluate quantitatively the effect of food intake on the drug - drug interaction between two new quinolones, ofloxacin or ciprofloxacin and sucralfate.

**Methods:**

The rats were orally administered new quinolones (5.3 mg/kg of ofloxacin or 10 mg/kg of ciprofloxacin) with or without 13.3 mg/kg of sucralfate under fasted or fed condition and plasma concentration profiles of new quinolones were monitored. To the fed group, standard breakfast used in human studies was pasted and administered at a dose of 8.8 g/kg.

**Results:**

The area under the plasma concentration - time curves (AUC_0–6_) of ofloxacin and ciprofloxacin under the fasted condition were significantly decreased to 28.8 and 17.1% by co-administration of sucralfate, respectively. On the contrary, sucralfate moderately decreased the AUC_0–6_ of ofloxacin and ciprofloxacin to 54.9 and 33.2%, respectively, under fed condition. The effects of sucralfate and food intake on the kinetics of ofloxacin in this study were well consistent with the results of previous clinical trial.

**Conclusions:**

The developed animal model quantitatively reproduced the effect of food intake on the drug - drug interaction between ofloxacin and sucralfate. The similar influences were observed for the drug - drug interaction between ciprofloxacin and sucralfate, suggesting that the extent of drug - drug interaction caused by chelation is generally attenuated by food intake.

## Background

Oral absorption of new quinolone antibiotics (NQs) is decreased by the coadministration of antacids containing polyvalent metal cations, which chelate with NQs. The extent of this drug-drug interaction (DDI) may be affected by food intake. Indeed, Kawakami et al. have reported in a clinical trial that sucralfate (AL) decreased the area under the plasma concentration - time curve (AUC) of oral ofloxacin (OFLX) remarkably to 38.7% under a fasted condition, but less so, to 68.7%, under a fed condition (30 min after meal) [1]. Therefore, the extent of DDI caused by physicochemical interactions such as chelation may be affected by food intake. However, no comprehensive and quantitative evaluation regarding the effect of food intake on this DDI has been reported and the clinical reports are quite limited.

The aims of this study were 1) to develop an animal model using rats to evaluate the effect of food intake on the extent of DDI caused by chelation in the gastrointestinal tract, and in consequence to mimic the DDI between OFLX and AL observed in clinical trials, and 2) to quantitatively evaluate the effect of food intake, using the developed model, on the DDI between AL and ciprofloxacin (CPFX), another NQ, in order to carefully evaluate the influence of food intake.

## Methods

### Animals

Male Sprague-Dawley rats (8 weeks) were purchased from Sankyo Labo Service Co. (Tokyo, Japan). They were allowed free access to food and water for 7 days and fasted overnight before the experiment. All animal experiments received approval from the Institutional Review Board and Animal Research Committee of the Keio University Faculty of Pharmacy. All experiments adhered to the university “Regulation for Animal Experimentation”, which is in accordance with the National Institutes of Health guide for the care and use of laboratory animals.

### In vivo drug and food administration experiment

The dosage of the drugs for the rats in this study was set based on the estimated concentration in the gastrointestinal tract in the clinical setting. Assuming the volume of the gastrointestinal tract of human (75 kg) to be 1000 mL, the concentrations of OFLX, CPFX and AL in the gastrointestinal tract are calculated to be 0.4, 0.75, and 1 mg/mL from the standard clinical doses of 400 mg [2], 750 mg [3] and 1 g [4], respectively. Since the volume of the gastrointestinal tract of rat is about 4 mL (13.3 mL/kg) [5], the doses of OFLX, CPFX and AL were calculated to be 5.3, 10 and 13.3 mg/kg, respectively. For OFLX or CPFX, Tarivid® tablet 100 mg (Daiichi Sankyo Co., Ltd., Tokyo, Japan) or Ciproxan® tablet 100 mg (Bayer Yakuhin, Ltd., Osaka, Japan) were administered after smashing. For AL, Ulcerlmin® Oral Suspension 10% (Chugai Pharmaceutical Co., Ltd., Tokyo, Japan) was diluted and administered. The standard breakfast (660 g, calcium content: 22.2 mg/100 g) consisted of 2 slices of bread, 2 pieces of bacon, 2 eggs, 240 mL of orange juice [6], and was prepared by blender to administer to rats in the fed condition. The dose of food was set to 8.8 g/kg for the rats, in proportion to that of humans (660 g/75 kg).

Under the fasted condition, each NQ was administered 5 min after the administration of AL or vehicle. Under the fed condition, the suspended food was given 5 min before the administration of AL or vehicle, which was followed by the oral administration of each NQ with a further 5 min interval. After all drug treatments were completed, the rats were kept in Bollman cages individually, and then 150 μL blood samples were collected from jugular vein cannula at 5, 10, 20, 30, 60, 120, 180 and 360 min after the oral administration of NQs.

Blood samples were immediately centrifuged at 3000×g for 10 min at 4 °C. The plasma was separated and stored at − 30 °C until analysis.

### Analysis of NQs by HPLC-UV method

A 250 μL aliquot of acetonitrile containing 0.2 μg/mL umbelliferone (Sigma-Aldrich, Tokyo, Japan) as the internal standard (I.S.) was added to 50 μL of each plasma sample and then the sample was centrifuged (4 °C, 1790×g, 10 min) after shaking for 2 min. After adding 200 μL purified water into each sample, the sample was vortexed for 2 min and centrifuged at 1790×g for 10 min at 4 °C. The supernatant (360 μL) was separated and dried under a gentle nitrogen stream at 40 °C. The residue was dissolved by 60 μL mobile phase and 20 μL of the sample was submitted to HPLC analysis as described below.

The HPLC system consisted of a pump (LC-10 AD, Shimadzu, Kyoto, Japan), a UV detector (SPD-10A, Shimadzu), and an octadecylsilane column (Cosmosil, 5C_18_-AR-II, 4.6 × 150 mm, Nacalai Tesque, Kyoto, Japan). The mobile phase consisted of 0.05 mM (NH_4_)_2_HPO_4_ including 2 mM EDTA (adjusted to pH 3.0 by phosphoric acid), methanol and acetonitrile (87: 7: 6, *v*/v) as previously reported [7], and was pumped at a rate of 1.5 mL/min. The absorbance of OFLX and CPFX measured at 294 and 278 nm, respectively. Concentrations were determined using the peak area ratio of the analyte (NQs) to I.S. (umbelliferone). The quantitation limit was 0.01 μg/mL for both OFLX and CPFX.

### Calculation of the pharmacokinetics parameters of the NQs

The AUC_0–6_ value of NQs was calculated by the linear trapezoidal method. The elimination rate constant (k_e_) was calculated from the slope of the elimination phase of the concentration profile. The elimination of drug from the systemic circulation was assumed to be first-order.

### Statistical analysis

As there are two factors, food intake and administration of AL, to be analyzed, the effects of these factors were assessed using two-way ANOVA (analysis of variance). Statistical difference in C_max_, AUC and k_e_ values were determined by two-way ANOVA followed by Bonferroni’s multiple comparison. A *P* value of less than 0.05 was considered statistically significant.

## Results

**Fig. 1:**
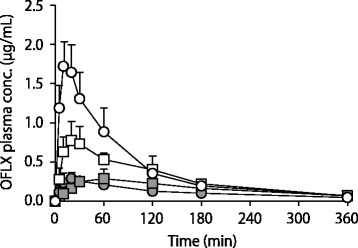
The plasma OFLX concentration profiles in rats after oral administration of OFLX at a dose of 5.3 mg/kg alone (open symbol) or with AL (13.3 mg/kg) (closed symbol) under fasted (circle symbol) or fed (square symbol) conditions. Each symbol represents the mean ± S.D. from 5 independent experiments

**Fig. 2:**
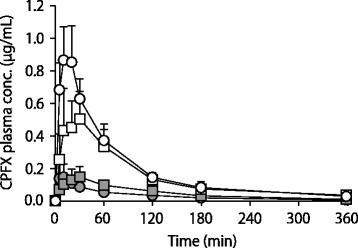
The plasma CPFX concentration profiles in rats after oral administration of CPFX at a dose of 10 mg/kg alone (open symbol) or with AL (13.3 mg/kg) (closed symbol) under fasted (circle symbol) or fed (square symbol) conditions. Each symbol represents the mean ± S.D. from 5 independent experiments

Under the fasted condition, the C_max_ and AUC_0–6_ values of OFLX were remarkably and significantly decreased to 17.2 and 28.8% respectively by coadministration of AL (Fig. 1, Table 1). In contrast, under the fed condition, the OFLX values were moderately and significantly decreased to 39.2 and 54.9% by AL (Fig. 1, Table 1). Similarly, the C_max_ and AUC_0–6_ values of CPFX were remarkably and significantly decreased to 17.3 and 17.1% respectively by AL under the fasted condition, and moderately and significantly decreased to 26.1 and 33.2% under the fed condition (Fig. 2, Table 2). Overall, under the fed condition, the extent of decrease in the C_max_ and AUC_0–6_ of the NQs by AL was smaller than the respective decrease under the fasted condition. The two-way ANOVA analysis revealed a significant interaction between coadministration of AL and food intake (*p* < 0.05). In other words, the influence of AL was differently evoked depending on the conditions of food intake.

**Table 1:** Effect of food intake on the pharmacokinetic parameters of OFLX after 5.3 mg/kg OFLX oral administration alone, and with 13.3 mg/kg AL in rats

Diet	AL	C_max_ (μg/mL)	AUC_0–6_ (μg∙h/mL)	k_e_ (h^− 1^)
Fasted	–	1.86 ± 0.26	2.53 ± 0.58	0.39 ± 0.10
	+	0.32 ± 0.06^*^	0.73 ± 0.11^*^	0.27 ± 0.14
Fed	–	0.82 ± 0.17^#^	1.82 ± 0.32^#^	0.45 ± 0.09
	+	0.32 ± 0.10^*^	1.00 ± 0.12^*^	0.32 ± 0.10

Data are mean ± SD, *n* = 5

^*^p < 0.05 vs respective OFLX alone group. ^#^*p* < 0.05 vs fasted OFLX group

**Table 2:** Effect of food intake on the pharmacokinetic parameters of CPFX after 10 mg/kg CPFX oral administration alone, and with 13.3 mg/kg AL in rats

Diet	AL	C_max_ (μg/mL)	AUC_0–6_ (μg∙h/mL)	k_e_ (h^− 1^)
Fasted	–	0.92 ± 0.19	1.15 ± 0.24	0.36 ± 0.11
	+	0.16 ± 0.08^*^	0.20 ± 0.07^*^	0.47 ± 0.37
Fed	–	0.59 ± 0.23^#^	0.90 ± 0.16^#^	0.25 ± 0.06
	+	0.15 ± 0.07^*^	0.30 ± 0.07^*^	0.39 ± 0.31

Data are mean ± SD, *n* = 5

^*^p < 0.05 vs respective CPFX alone group. ^#^*p* < 0.05 vs fasted CPFX group

## Discussion

In this study, we evaluated the effects of food intake on the extent of DDI between NQs and AL in the gastrointestinal tract using an animal model. As a result, the decrease in the AUC value of OFLX by AL was 28.8% and 54.9% in the fed and fasted conditions, respectively, demonstrating that the inhibition of OFLX intestinal absorption by AL was attenuated by food intake. A previous clinical study showed the AUC value of OFLX decreased by AL up to 38.7% under a fasted condition, but only to 68.7% at 30 min after breakfast [1]. The influence of AL on the AUC value of OFLX under the fasted and fed conditions was consistent with those results in the present study. The doses of NQs and AL in this study were determined based on their estimated clinical concentration in the gastrointestinal tract. Therefore, the doses of OFLX (5.3 mg/kg) and AL (13.3 mg/kg) also correspond to the conditions of the clinical study in Lehto et al. [8], where the AUC value of OFLX was reported to be decreased to 38.9% by AL under a fasted condition. As the decrease in the AUC value of OFLX under a fasted condition by AL reported by Kawakami et al. and Lehto et al. is almost quantitatively consistent to the observation in this study (Table 1), the dose-setting of the present rat model based on the volume of the gastrointestinal tract may overcome the interspecies differences between rats and humans; after quantitatively evaluating the extent of DDI in the clinical settings. Meanwhile, the influence of food intake on the AUC value after the administration of OFLX and AL was also consistent with the results of the clinical trial [1]. Therefore, the present approach using rats administered a scale-downed dose of smashed standard breakfast is considered to be appropriate for the evaluation of the effect of food intake on the extent of DDI in the gastrointestinal tract. Overall, the animal model, which enabled us to quantitatively evaluate the influence of food intake on the extent of DDI in the gastrointestinal tract, was successfully developed in this study.

For CPFX, no studies have reported the effect of food intake on the extent of DDI with AL. Our present findings using the developed animal model strongly suggest that food intake attenuates DDI between AL and CPFX, as well as that between AL and OFLX, in the clinical setting. Although further experiments may be required, the current findings for this DDI may be applied to various other NQs.

Although the exact mechanism for the attenuation of the DDI by food intake remains to be investigated, there are some possible mechanisms: 1) food increased the volume of digesta, diluted the gastrointestinal concentration of drugs, and decreased the chance of interplay between NQs and AL, 2) the digesta adsorbed NQs and/or AL to block chelation, 3) food increased the viscosity of digesta, decreasing mixing. Further investigations are required to clarify the mechanism(s) responsible for this food-intake effect, as well as the effect of type of foods, e.g. high-fat food, on the extent of DDI.

In this study, the absorption of CPFX was more potently inhibited by AL than that of OFLX (Tables 1 and 2). This finding is also consistent with a clinical study showing that the influence of AL on the absorption of NQs varies among NQs and CPFX is more vulnerable to AL than OFLX [9]. This difference can be also explained by the in vitro binding data. The binding constant of CPFX with Al^3+^ is 8.434 × 10^4^ M^− 1^, while that of LVFX, an optically active isomer of OFLX, is weaker at 2.459 × 10^4^ M^− 1^ [10].

The DDI caused by chelation is not limited to the cases of NQs and AL. Tetracycline antibiotics are typical examples of interaction with various metal cations [11]. Other examples include cephem antibiotics that quite selectively bind to a ferrous preparation to lead to decreased absorption [12]. No clinical studies have been conducted to elucidate the effect of food intake on the extent of DDI for these drugs. However, it is likely that food intake also attenuates these interactions as well because they share the same mechanism, i.e. chelation in the gastrointestinal tract.

One of the limitations of this study is the interspecies differences, which should be taken into consideration to interpret our results. The two-way ANOVA analysis showed statistically significant interaction between two factors, the food intake and coadministration of AL, showing that the extent of this DDI was attenuated by food intake, being consistent with the clinical observation. On the other hand, the effect of food itself was different between animal model and humans; in the absence of AL, the food decreased the C_max_ and AUC values of OFLX in rats but not in humans [1]. Food intake may delay the gastric empty rate and OFLX concentration in the gastrointestinal tract, probably in both rats and humans. However, as the permeability surface in rats is far smaller than that in humans, the absorption kinetics is conceivably more susceptible to the decrease in OFLX concentration in the gastrointestinal tract. The conditions of the present animal model may need to be improved so that it allow us to quantitatively mimic the effects of food intake on the absorption profile of a drug, as well as that on DDI.

## Conclusion

In this study, the developed animal model quantitatively reproduced the effect of food intake on the DDI between OFLX and AL. A similar influence was observed for the DDI between CPFX and AL. This study suggested that the extent of DDI caused by chelation is generally attenuated by food intake. In the clinical setting, the extent of the DDI after coadministration of these types of drugs under the fed condition may be decreased compared with that under the fasted condition.

## Abbreviations

AL: Sucralfate
AUC: The area under the plasma concentration - time curve
CPFX: Ciprofloxacin
DDI: Drug - drug interaction
NQs: New quinolone antibiotics
OFLX: Ofloxacin
